# Public health round-up

**DOI:** 10.2471/BLT.16.011016

**Published:** 2016-10-01

**Farewell to malaria**

**Sri Lanka was certified by the World Health Organization (WHO) last month as having eliminated malaria, a life-threatening disease that had long affected the island country. For the past three-and-a-half years, no locally transmitted cases have been recorded. The picture shows a mother and her child under a mosquito net in a refugee camp in north central Sri Lanka in 2003.**

**Figure:**
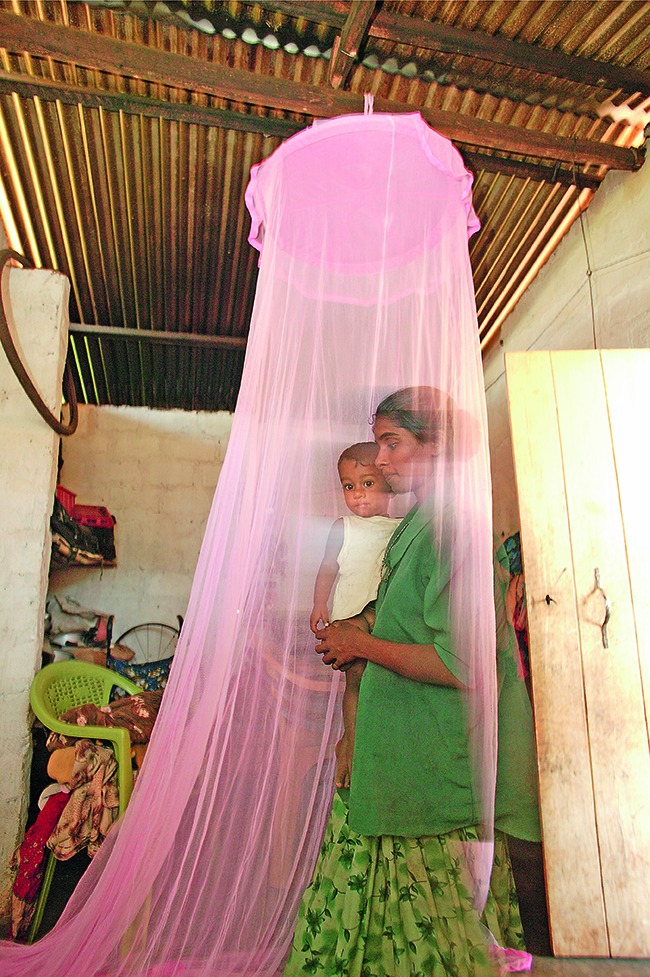
The Global Fund/John Rae

## Health jobs for youth and women 

Governments need to create more jobs in the health sector to boost their economies and make progress in achieving their health goals, according to the High-Level Commission on Health Employment and Economic Growth. 

In its final report released last month, the commission, co-chaired by President François Hollande of France and President Jacob Zuma of South Africa, made 10 recommendations to address the pressing need for health workers in many countries. 

The report proposes changes in health employment, health education and health service delivery policies and stresses that creating jobs in the health sector should be considered an investment, not a cost. 

Populations are ageing and there are increasing rates of noncommunicable diseases. 

To address these and other challenges, an estimated 40 million more health workers will be created globally to address the growing demands for health and social care by 2030, but by the same date a shortfall of 18 million health workers is projected, primarily in low- and middle-income countries.

“To address this imbalance, the Commission’s report articulates a powerful narrative that views investments in the health workforce as contributing to more equitable health care, the creation of millions of decent jobs, and the promotion of economic growth that is inclusive, especially for youth and women,” said Dr Margaret Chan, Director-General of WHO told the Regional Committee for Europe last month, 

Chan, who is co-vice-chair of the commission, along with Guy Ryder, Director-General of the International Labour Office and Angel Gurría, Secretary-General of the Organisation for Economic Co-operation and Development agreed to convene relevant stakeholders by the end of 2016 to develop a five-year implementation plan for the 10 recommendations.

The report cites evidence that the returns on investment in health are estimated to be 9 to 1 and that about one quarter of economic growth between 2000 and 2011 in low- and middle-income countries is estimated to have resulted from improvements in health.

The commission, set up by the United Nations Secretary-General Ban Ki-moon in March, released its report at the United Nations General Assembly on 20 September. 

## WHO’s clean air campaign

WHO is due to launch its new BreatheLife clean air campaign at Habitat III, the United Nations conference on housing and sustainable urban development in Quito, Ecuador this month. 

The campaign, a collaboration with the United Nations Environment Programme-hosted Climate and Clean Air Coalition to Reduce Short-lived Climate Pollutants, aims to promote health as a central part of the new urban development agenda and is supported by the governments of Norway, Chile and other partners.

Air pollution is a major risk factor for heart disease, stroke, chronic obstructive pulmonary disease and lung cancer. It also increases the risks for acute respiratory infections and exacerbates asthma. 

Reducing air pollution has double benefits: it reduces carbon emissions and other toxic air particles that lead to 6.5 million deaths each year and it helps to reduce global warming to less than 2 degrees Celsius in line with the 2015 Paris Agreement on climate change.

Hundreds of people from governments, civil society, science and the private sector are set to gather at the Habitat III conference from 17 to20 October to agree on an urban development agenda to make cities economically vibrant, environmentally sustainable and socially equitable. 

The conference is held every 20 years; the last event took place in Istanbul, Turkey in 1996. 

## Emergency in Nigeria

WHO, with its partners, is scaling up its emergency health response to the humanitarian crisis in north eastern Nigeria, where some 3.7 million displaced people are in desperate need of health services.

A recent WHO rapid assessment of 56 health facilities in the Nigerian state of Borno found dire shortages of medicines and other supplies, especially for paediatric, emergency obstetric and antenatal care.

The health facilities are struggling to provide services to some 1.3 million displaced people in Borno state, formerly held by militant insurgency groups, the assessment found.

Stronger disease surveillance is urgently needed to allow for the early detection of infectious diseases. 

WHO is working with the Government of Nigeria and partners to quickly re-establish and strengthen early warnings for disease outbreaks to allow for more rapid responses. 

To prevent potential outbreaks, WHO has supported the establishment of an early warning alert and response system in the 56 health facilities. 

WHO also provided training on how to use the system for more than 80 health professionals.

“Our immediate goal is to reduce the rates of death and disease by rapidly scaling up life-saving health services in spite of continued insecurity,” said Rob Holden, Nigeria Emergency Coordinator from WHO’s Health Emergencies Programme.

A third round of emergency polio vaccination was completed in northern Nigeria last month after an outbreak of the disease was declared a national and regional public health emergency.

The third vaccination round covered 11 states in northern Nigeria that are at high risk of renewed polio transmission, as well as other states. A fourth round is planned for later this month. 

WHO is working closely with the federal and state ministries and with many health partners to quickly dispatch medicine and supplies to the vulnerable population in Borno state.

http://www.who.int/emergencies/nigeria/

## Yellow fever vaccination in Kinshasa

More than seven million people were immunized against yellow fever with reduced doses of the vaccine in Kinshasa, the capital of the Democratic Republic of Congo (DRC), in a huge emergency vaccination campaign.

The campaign was rolled out within the space of two weeks in August to curb the spread of the disease before the onset of the rainy season in the southern African country in September.

The health ministry led the campaign with support from WHO and global partners, based on advice from WHO’s Strategic Advisory Group of Experts on Immunization (SAGE) that fractionation could be used as a short-term emergency measure to reach more people, given the shortage of vaccine supplies.

The SAGE cited evidence that a fifth of a standard vaccine dose provides protection against the disease for at least 12 months.

About 10 million syringes were shipped to the country and more than 40 000 vaccinators were trained in fractionation.

The campaign came months after a yellow fever outbreak started in neighbouring Angola in December 2015 and spread to DRC earlier this year.

http://www.who.int/emergencies/yellow-fever/

**Cover photo**

This month’s cover photo shows a mother holding her four-month-old baby born with microcephaly in Recife, Brazil. The growing Zika epidemic in the Americas and its link with birth defects, such as microcephaly, and with Guillain–Barré syndrome was declared a public health emergency in February 2016.

**Figure:**
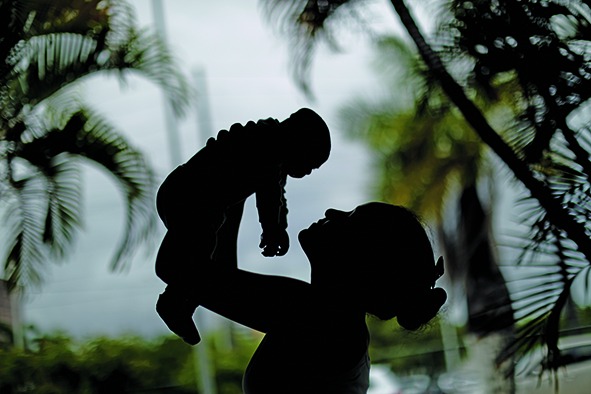
© UNICEF/ Ueslei Marcelino

## Financing health in Africa

Countries in the WHO African Region need to change the way they allocate and use public funds for health if they are to move towards universal health coverage, according to a new WHO report.

The report, entitled *Public financing for health in Africa: from Abuja to the SDGs*, identifies four key areas of concern: prioritization of health budget, funding inconsistency, budget underspends and misallocation of resources.

“Too often public resources are fragmented, poorly distributed and inefficiently used,” said Dr Marie-Paule Kieny, Assistant Director-General for Health Systems and Innovation at WHO headquarters and Dr Matshidiso Moeti, Regional Director for WHO's Africa Region in the foreword of the report.

“As a result they do not benefit the people who need them most,” they said.

The report was released at the end of August, a few days before the World Bank and the Global Fund to Fight AIDS, Tuberculosis and Malaria agreed to invest US$ 24 billion for universal health coverage in Africa.

The joint pledge was announced at the 6th Tokyo International Conference on African Development in Nairobi, Kenya.

In 2001, African leaders pledged that they would allocate at least 15% of their annual budget to the health sector. But, according to the new report, average annual public expenditure on health in the region in 2014 was only 10% of total public spending and less than half of that goes to priority health services, such as primary care and maternal and child health.

The report reviews the main trends in public financing for health over the past 15 years in the African region.

It argues that increasing public budgets is not enough to make progress towards universal health coverage, but that appropriately targeted health budget allocations and full execution of public health budgets and improved efficiency are essential.

http://www.who.int/health_financing/documents/public-financing-africa/

## UN report on access to medicines

A binding international treaty on research and development that delinks prices from research and development (R&D) costs is needed to make medicines and other health technologies more accessible, according to a report by the United Nations Secretary General High-Level Panel on Access to Medicines. 

In its report**that was**submitted to the UN Secretary General on 12 September, the panel called for the creation of new bodies to take this and its other recommendations forward.

The panel urged governments to make better use of World Trade Organization rules to increase access to affordable medicines and increase transparency in the costs of research and development. 

United Nations Secretary-General Ban Ki-moon convened the panel in November 2015 “to review and assess proposals and recommend solutions for remedying the policy incoherence between the justifiable rights of inventors, international human rights law, trade rules and public health in the context of health technologies.” 

http://www.who.int/phi/implementation/ip_trade/high-level-panel-access-med/

**Looking ahead**

**21–24 November – 9th Global Conference on Health Promotion, **Shanghai, China

**14–20 November – World Antibiotic Awareness Week**

**1 December – World AIDS Day**

